# Grape seed extract supplement increases bone callus formation and mechanical strength: an animal study

**DOI:** 10.1186/s13018-019-1251-5

**Published:** 2019-07-05

**Authors:** Murat Gurger, Erhan Yilmaz, Seval Yilmaz, Gokhan Once, Mustafa Konuk, Emre Kaya, Yakup Say, Gokhan Artas, Hakan Artas

**Affiliations:** 1 Department of Orthopaedics and Traumatology, Faculty of Medicine, Firat University, 23119 Elazig, Turkey; 2 Department of Biochemistry, Faculty of Veterinary Medicine, Firat University, 23119 Elazig, Turkey; 3 Department of Orthopaedics and Traumatology, Tatvan State Hospital, 13200 Bitlis, Turkey; 4 Department of Metallurgical and Materials Engineering, Tunceli University, 62000 Tunceli, Turkey; 5 Department of Medical Pathology, Faculty of Medicine, Firat University, 23190 Elazig, Turkey; 6 Department of Radiology, Faculty of Medicine, Firat University, 23190 Elazığ, Turkey

**Keywords:** Grape seed, Proanthocyanidin, Antioxidant, Fracture healing, Biomechanics

## Abstract

**Background:**

The positive effects of grape seed proanthocyanidin extract (GSPE) on bone health, which is a potent antioxidant, are known but its effects on fracture healing are not sufficiently covered in the literature. This study aims to investigate the effects of GSPE on fracture healing and biomechanics of healing bone.

**Materials and methods:**

Sixty-four adult Wistar-Albino male rats were divided into 8 groups of 8 animals in each group. Osteotomy was performed to the right femurs of all groups except the negative control (G1) and positive control (G2) groups, and intramedullary Kirchner wire was used for fixation. GSPE was given to half of the rats (G2-G4-G6-G8) 100 mg/kg/day by oral gavage. The rats were sacrificed on the tenth (G3–G4), twentieth (G5–G6), and thirtieth (G1–G2–G7–G8) days, respectively, and histopathological, radiological, and biomechanical examinations were performed.

**Results:**

Histopathological examination of the specimens from the callus tissues revealed that bone healing was more prominent in the groups supplemented with GSPE (G4, G6, G8). There was a statistically significant improvement in radiological recovery scores and callus volumes in groups with GSPE. When biomechanical strengths were evaluated, it was found that GSPE increased bone strength not only in fracture groups but also in the positive control group (G2).

**Conclusions:**

As a result, this study showed that GSPE, a potent anti-oxidant, had a positive effect on bone healing and improved mechanical strength of the healing bone.

## Background

Bone fractures are one of the most common injuries. Despite optimal treatment approaches, undesirable results occur in about 10% of bone fractures. Treatment of non-union fractures is a painful process accompanied by clinical, social, and economic problems [1]. Fracture union is a dynamic process that results in bone remodeling, and understanding the physiology of this process is important to achieve optimal results in clinical practice. An accurate osteosynthesis allows many phases to begin in the process of fracture healing. This process is a complex process involving the organization of the fracture hematoma, fibroplasia, chondroplasia and osteoplasia, and ultimately new bone formation. This physiological process does not always function flawlessly. For example, free oxygen radicals and products are formed in relation to the damaged blood supply in the fracture site. This is a local form of oxidative stress [2]. Oxidative stress can be defined as a chain of oxidative events that causes the production of reactive oxygen species that cause tissue damage. The first 3 days of fracture healing is similar to the ischemia period of ischemia-reperfusion event and no oxidative stress damage occurs in this period. Then, in the stage of callus formation, inflammatory cells that come with newly formed capillaries increase the production of free oxygen radicals. If increased free oxygen radicals in the fracture zone exceed the natural antioxidant defense mechanisms, oxidative damage, similar to the reperfusion injury seen in other tissues, may occur in the broken bone [3]. In such cases, an antioxidant uptake is a rational approach to suppress the destructive effects of free oxygen radicals and to improve fracture healing [2]. Grape seed proanthocyanidin extract (GSPE) is a naturally occurring polyphenolic compound from *Vitis vinifera* seeds and GSPE has a wide range of biological properties against oxidative stress [4]. Besides its free radical scavenging and antioxidant functions, GSPE has numerous roles, such as vasodilator, antiallergic, immunostimulator, anti-inflammatory, cardioprotective, antiviral, antibacterial, and anticarcinogen activities. In previous studies, it has been reported that GSPE may reduce lipid peroxidation, capillary permeability and fragility, platelet aggregation and regulate phospholipase A2, cyclooxygenase and lipoxygenase enzyme activities [5, 6]. GSPE contains the following components: 15% (+) -catechin and (−) -epicatechin, 80% (−) -epicatechin 3-O-gallate, dimers, trimers, tetramers, and their gallates; and 5% pentamers, hexamers, heptamers, and their gallates [7]. The association between flavonoids and bone health, such as genistein (4′,5,7-trihydroxyiso-flavone) and daidzein (4′,7-dihydroxyisoflavone) has been demonstrated in previous studies [8, 9]. GSPE, which is a flavonoid, is also known to play a role in the regulation of bone homeostasis [4]. Usage of GSPE has shown to increase bone density and strength in addition to its protective and curative effects in osteoporosis, osteonecrosis, and inflammatory autoimmune arthritis [5, 10–12]. However, its effects on fracture healing are not sufficiently available in the literature. From this point of view, we aimed to investigate the effects of GSPE on fracture healing.

## Materials and methods

### Animals, grouping, and treatment

Sixty-four adult Wistar-Albino male rats (age, 2–3 months; weight, 350 ± 50 g) were maintained at room temperature (25 °C), under 12/12 h of light/dark cycle, providing free access to food and water. Animals were fed with standard rodent diet. Surgical treatment and postoperative care were applied to all animals except control groups. The groups are as shown in Table 1.

**Table 1:** Sample groups

Groups	Application
G1 (*n* = 8)	No fracture, standard diet (negative control)
G2 (*n* = 8)	No fracture, GSPE supplement for 30 days (positive control)
G3 (*n* = 8)	Post fracture, standard diet for 10 days
G4 (*n* = 8)	Post fracture, GSPE supplement for 10 days
G5 (*n* = 8)	Post fracture, standard diet for 20 days
G6 (*n* = 8)	Post fracture, GSPE supplement for 20 days
G7 (*n* = 8)	Post fracture, standard diet for 30 days
G8 (*n* = 8)	Post fracture, GSPE supplement for 30 days

Grape seed proanthocyanidin extract (Solgar, Leonia; NJ, USA) was dissolved in 1% carboxymethyl cellulose and then it was applied to rats in groups G2, G4, G6, and G8 at a dose of 100 mg/kg/day by oral gavage.

### Surgical method and tissue preparation

The rats were anesthetized with intraperitoneal ketamine (40 mg/kg Ketalar, Eczacibasi, Istanbul, Turkey) and xylazine (5 mg/kg Rompum, Bayer, Leverkusen, Germany). The osteotomy was performed by multi-drilling technique on the middle 1/3 of the femur shaft with an approximately 0.5 cm incision from the right thigh lateral under sterile conditions [13]. The medial parapatellar approach was then introduced to the knee area of the same side; the knee was flexed and following the reduction of the fracture, a 1 mm Kirchner wire was applied retrograde for intramedullary fixation from the intercondylar region. The incisions were appropriately closed with 3.0 Vicryl rapid. Animals were given full weight bearing and unlimited movement post-anesthesia. For pain control during the postoperative period, Buprenorphine was administered subcutaneously 0.05 mg/kg twice daily for 3 days. Cefazolin as a prophylactic antibiotic was administered intraperitoneally at a dose of 30 mg/kg. According to the treatment protocol of their group, radiographic examinations of animals completing the test period were performed. Then, rats were sacrificed with the overdosage of sodium pentobarbital (400 mg/kg), and the right femurs were dissected. After removal of the Kirshner wire used for fixation, bones were sent to the laboratory for histopathologic tests in a 10% buffered formaldehyde solution, whereas femurs were maintained at − 20 °C for biomechanical tests.

### Histopathologic evaluation

Bone tissues were put in 10% buffered formaldehyde solution for 48 h and then transferred into decalcification solution (facepath decalcification solution). After daily controls were performed and adequate softening was achieved, routine histopathological follow-up was performed. Four micrometer sections were taken from prepared paraffin blocks and Hematoxylin-Eosin staining was applied. The dyed preparations were evaluated with a light microscope (Leica DM500) and visualized by a digital camera (Leica DFC295). Evaluation is performed by using the scoring system developed by Huo et al. [14] (Table 2).

**Table 2:** Histopathological scoring used in the evaluation of fracture healing

Score	Histological findings in the fracture area
1	Fibrous tissue
2	A large amount of fibrous tissue and a small proportion of cartilage tissue
3	An equal amount of fibrous tissue and cartilage tissue
4	Cartilage tissue
5	A large amount of cartilage tissue and a small amount of immature (woven) bone tissue
6	Equal ratio of cartilage tissue and immature bone tissue
7	A large amount of bone tissue and a small proportion of cartilage tissue
8	Fully immature (woven) bone
9	Immature bone and a small amount of mature bone
10	Mature (lamellar) bone

### Radiological evaluation

For radiological evaluation, anteroposterior and lateral radiographs of the right femurs of the rats were taken. The fracture healing in fractured groups (G3,4,5,6,7,8) was evaluated by a radiologist, who did not know the details of the study, by using the radiological scoring system defined by Lane et al. [15] (Table 3). The fracture site was scanned with 64-detector row scanner (Aquilion; Toshiba, Tokyo, Japan) with the following parameters: 80 kV, variable mA (sure exposure), 0.5-mm section thickness, 0.35 s per rotation, and 1.1 beam pitch. Evaluation of callus volume was performed using a Digital Imaging and Communications in Medicine (DICOM) viewer (OsiriX MD 9.0, Pixmeo SARL, Berne, Switzerland) on coronal sections. The fracture line was determined in the longitudinal sections of the callus and the area under and above 3.2 mm was scanned [16].

**Table 3:** Lane-Sandhu radiological scoring system

Category	Points
Bone formation (the highest score is 4)	
No evidence of bone formation	0
Bone formation occupying 25% of the defect	1
Bone formation occupying 50% of the defect	2
Bone formation occupying 75% of the defect	3
Full gap bone formation	4
Union (the highest score is 4)	
Non-union	0
Possible union	2
Radiographic union	4
Remodeling (the highest score is 4)	
No remodeling	0
Remodeling of the intramedullary channel	2
Full remodeling of the cortex	4
Sum of radiographic scores	12

### Biomechanical evaluation

**Fig. 1:**
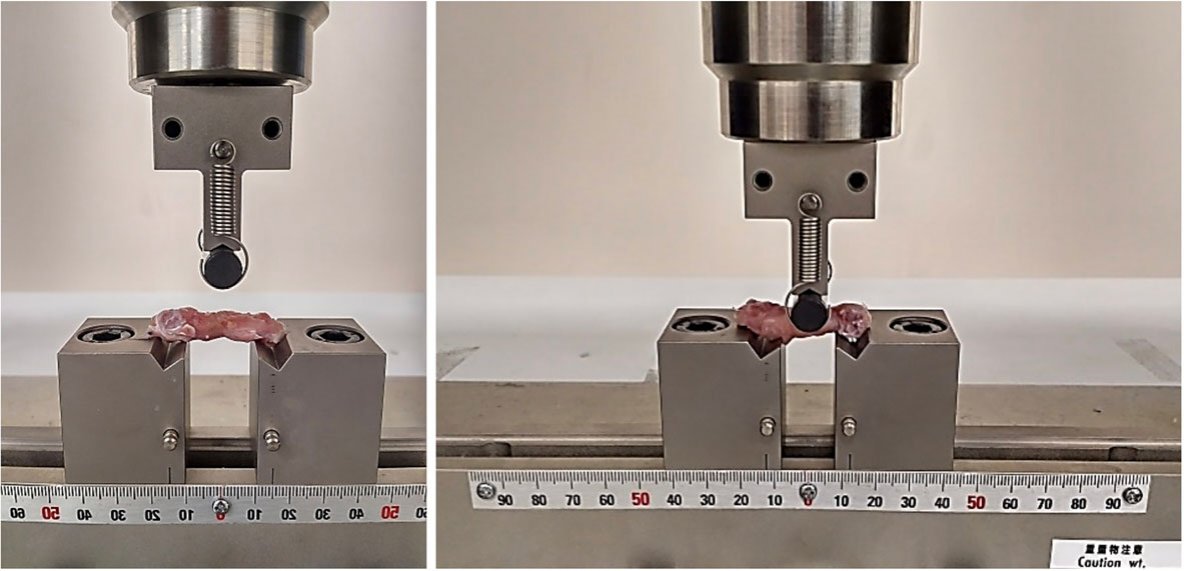
3 PB tests

The femurs were tested immediately after thawing and kept moist during the tests. 3-point bending tests (3 PB) were performed on a universal test machine (Shimadzu AG-IC 100 kN, Japan) at a test speed of 5 mm/min for all groups in the study (Fig. 1).

**Fig. 2:**
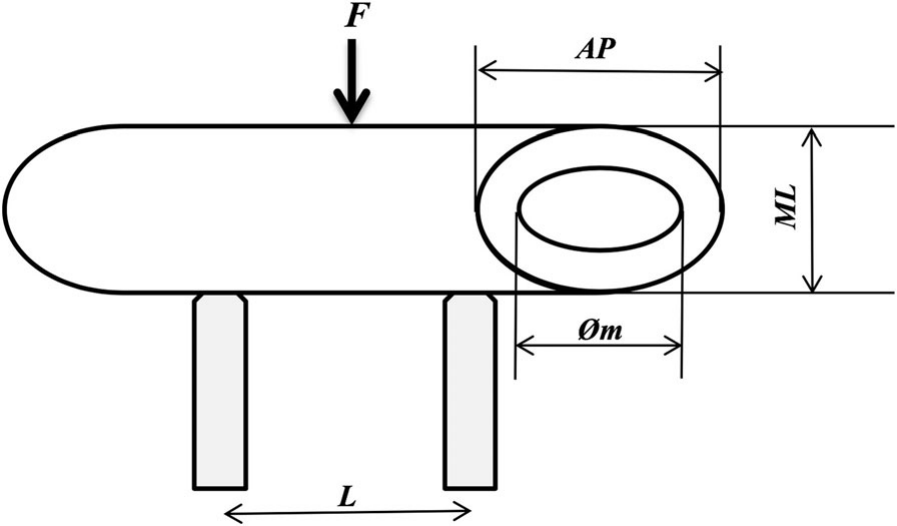
Guide to sample measurements used in calculations

After the biomechanical measurement, force-distance values were obtained. The anteroposterior “AP” (mm), mediolateral “ML” (mm) distances along with L distance between two beds (mm) were recorded by measuring the fracture line formed after tests as shown in Fig. 2. Medullary diameter “*m*” was fixed as 2 mm. The cross-sectional area “*A*” (mm^2^) of the bone structure in the sample groups was calculated using Eq. 1, and the moment of inertia of the section “*I*” (mm^4^) using Eq. 2.

1 $$ A=\pi\ \left(\frac{\left(\mathrm{AP}\ x\ \mathrm{ML}\right)-{m}^2}{4}\right) $$

2 $$ I=\frac{\pi }{2}\ \left(\left(\frac{\mathrm{AP}}{2}\right){\left(\frac{\mathrm{ML}}{2}\right)}^3-{\left(\frac{m}{2}\right)}^4\right) $$

Equation 3 was used to determine “Mb” bending moment (Nmm), while Eq. 4 was used to determine “*W*” strength module (mm^3^). “σ_max_” maximum stress (N/mm^2^) was calculated with the obtained values (Eq. 5).

3 $$ {M}_b=\frac{F_{\mathrm{max}}\ x\ L}{2} $$

4 $$ W=\frac{I}{\left(\mathrm{ML}/2\right)} $$

5 $$ \sigma =\frac{M_e}{W} $$

Finally, the elastic module “*E*” (MPa) was calculated by Eq. 6 based on maximum force “*F*_max_” (*N*), deflection “δ” (mm), and the moment of inertia “*I*” (mm^4^) of the section.

6 $$ E=\frac{F_{\mathrm{max}}\ x\ {L}^3}{48\ x\ I\ x\ \delta } $$

### Statistical analysis

After data collection, statistical analyses were performed by using SPSS 21.0 package program. Kolmogorov-Smirnov and Shapiro-Wilk normality tests were used to determine the distribution of continuous variables. Kruskal-Wallis test and *H* test along with post-hoc test were used to compare more than two independent groups that did not comply with normal distribution, while Man-Whitney *U* test was used to determine the relationship between two independent groups. Numerical data were expressed as mean ± standard deviation and median (min-max), qualitative data as percentages. *p* < 0.05 was considered significant.

## Results

### Histopathological results

**Fig. 3:**
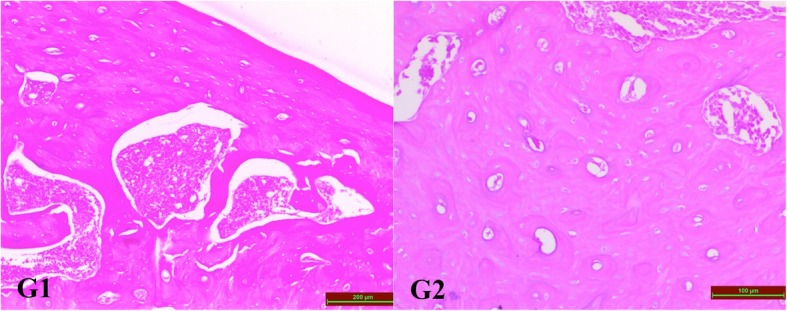
Micrographs of sections from G1 and G2 groups. Bone tissue sections in the negative control (G1) and positive control (G2) groups showed normal bone appearance

**Fig. 4:**
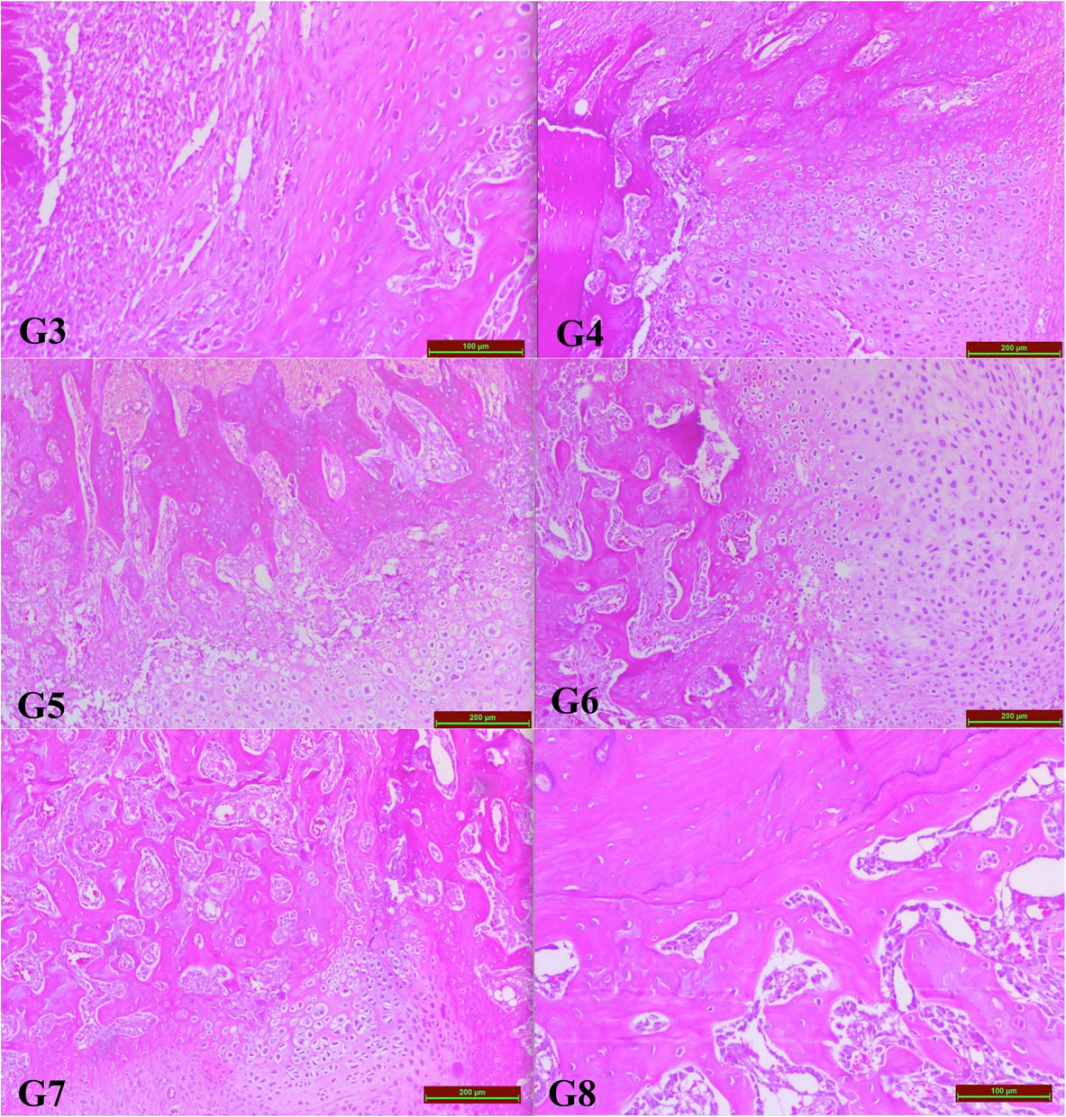
With time, healing of the fracture was observed in the examination of the samples taken from the callus tissue; 10 days after (G3, G4), 20 days after (G5, G6), and 30 days after (G7, G8) the fracture was introduced. However, bone healing was more evident in the groups given GSPE (G4, G6, G8)

As a result of the evaluation of hematoxylin and eosin staining according to Huo score under light microscopy; bone tissue was normal in G1 (negative control) and G2 (positive control) groups (Fig. 3). The difference between the Huo scores of the groups given GSPE and the scores of the groups not-given GSPE was statistically significant (Table 4), and it was observed that the bone healing in the groups given GSPE was better (Fig. 4).

**Table 4:** Median (min-max) values of Huo scores of groups

Groups not-given GSPE	Groups given GSPE	*p*
G1 = 10 (10–10)	G2 = 10 (10–10)	> 0.05
G3 = 3.5 (3–4)	G4 = 5.5 (5–6)	< 0.05
G5 = 5.0 (4–6)	G6 = 6.5 (6–7)	< 0.05
G7 = 7.5 (7–8)	G8 = 8.5 (8–9)	< 0.05

### Radiologic results

**Fig. 5:**
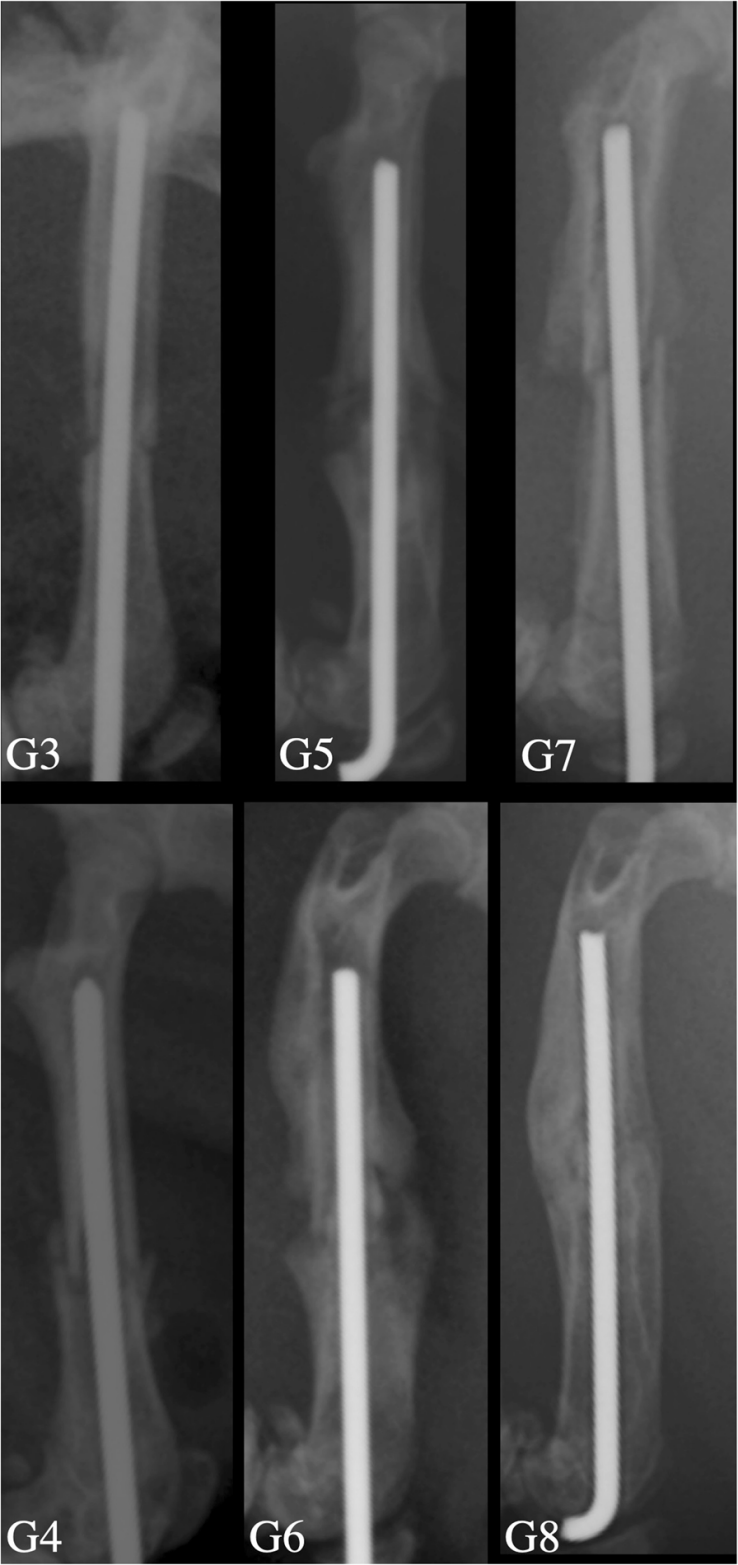
Radiographs of the samples of the groups in the 10th (G3, G4), 20th (G5, G6), and 30th (G7, G8) days. It was observed that the radiological improvement in the GSPE-treated groups (G4, G6, G8) was better than the non-GSPE groups (G3, G5, G7)

As shown in Fig. 5, radiological bone healing was higher in groups given GSPE. The difference between the results was statistically significant (Tables 5 and 6). When we look at the relationship between fracture healing and time, it was observed that there was a statistically significant difference between the first 10 days and 30 days (Table 7).

**Table 5:** Lane-Sandhu scores of the fracture introduced groups

Groups not-given GSPE*	Groups given GSPE*	*p*
G3 = 3.0 (1–4)	G4 = 5.0 (4–7)	< 0.001
G5 = 4.0 (3–5)	G6 = 7.0 (6–8)	< 0.001
G7 = 6.0 (5–8)	G8 = 8.0 (7–9)	< 0.001

*Median (min-max)

**Table 6:** Callus volumes of the fracture introduced groups

Groups not-given GSPE*	Groups given GSPE*	*p*
G3 = 0.024 (0.004–0.030)	G4 = 0.062 (0.058–0.078)	< 0.001
G5 = 0.035 (0.030–0.046)	G6 = 0.108 (0.078–0.150)	< 0.001
G7 = 0.050 (0.046–0.056)	G8 = 0.194 (0.155–0.280)	< 0.001

*Median (min-max)

**Table 7:** The change of fracture healing over time*

	10	20	30	*χ²*	*p*
Lane-Sandhu score	4.5 (1–6)	5.0 (4–7)	8.0 (4–9)^a^	12.857	0.002
Callus volume (cm^3^)	0.044 (0.004–0.078)	0.062 (0.03–0.15)	0.105 (0.05–0.28)^b^	10.184	0.006

^a^Comparing Lane-Sandhu score from day 10 to day 30, *p* = 0.001

^b^Comparing callus volume on day 10 to day 30, *p* = 0.004

*Median (min-max)

### Biomechanical results

**Fig. 6:**
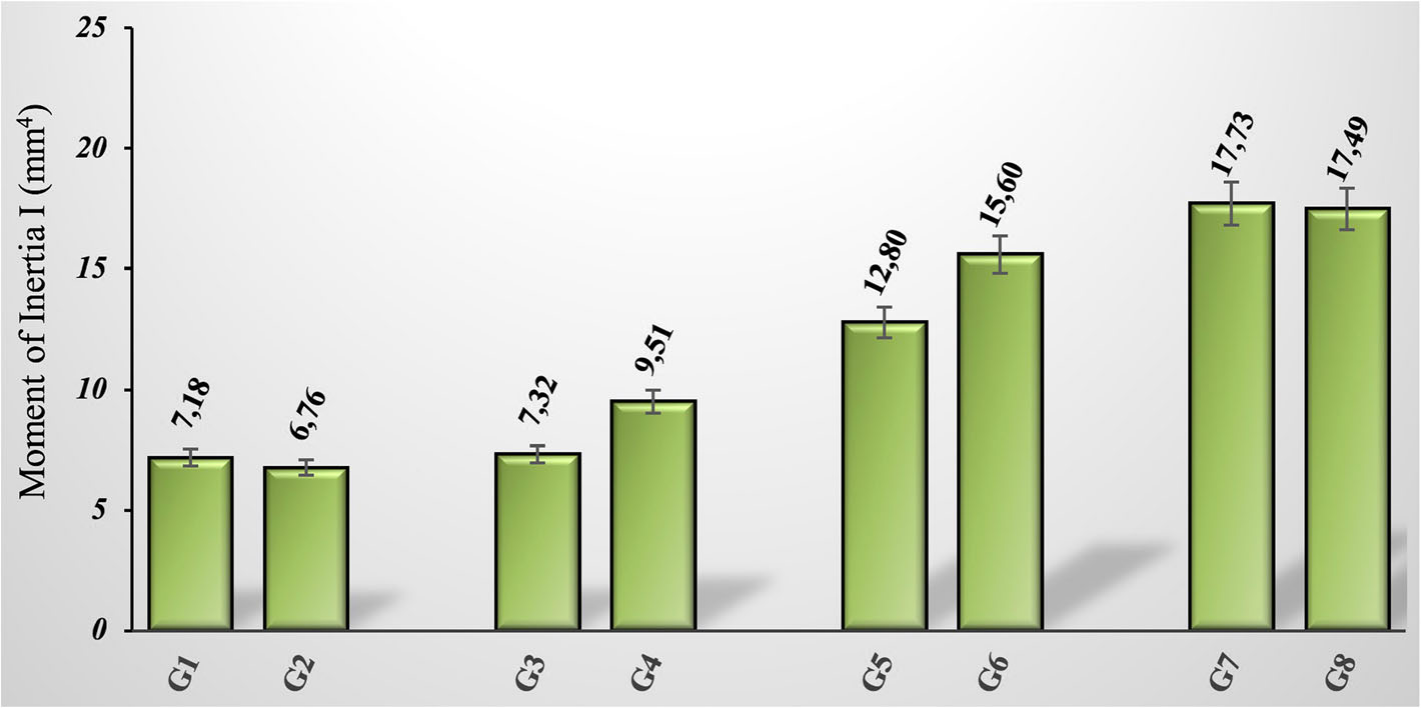
Calculated moment of inertia values

Although medullar diameter was fixed as 2 mm for all sample groups, AP and ML values differed due to the recovery time of osteotomy. It was found that the moment of inertia values increased with the increase in the healing time of the fractured bones (Fig. 6). The GSPE given groups are compared with the groups that are not given GSPE; especially on the 10th and 20th days, it was observed that the values of the moment of inertia increased in the groups given GSPE. Although the inertia moment value of the G7 sample was calculated to be slightly higher than the G8 sample at day 30, it can be said that the GSPE supplement generally increases AP and ML values in the fracture healing area, meaning that the GSPE supplement increases the callus formation in the fracture area.

**Fig. 7:**
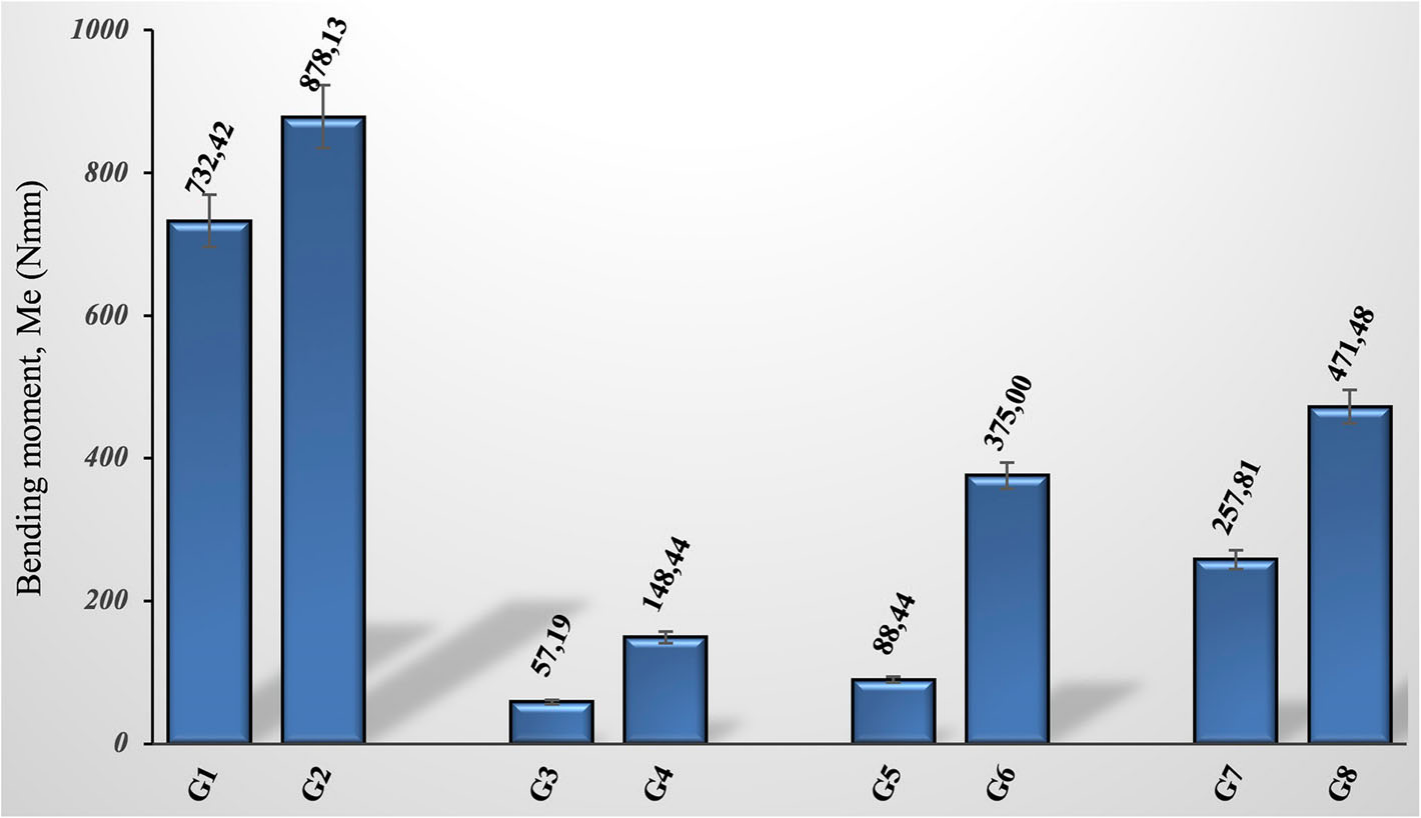
Calculated bending moment values

Figure 7 shows the bending moment values. The results show that in all groups, GSPE given groups had higher bending moment values compared to other groups. The mean bending moment of the negative control group (G1) was measured as 732.42 Nmm, whereas in the positive control group (G2) this value was measured as 878.13 Nmm by 20% increase. To evaluate the effect of GSPE supplement on the bending moment on fractured bones; for the 10th, 20th, and 30th days, we can see that the bending moment increases by approximately 160%, 324%, and 83% respectively. The main reason for these severe differences in bending moment values is the change in maximum force values. Similarly, as expected, bending moment values increase with increasing time of recovery (Fig. 7).

**Fig. 8:**
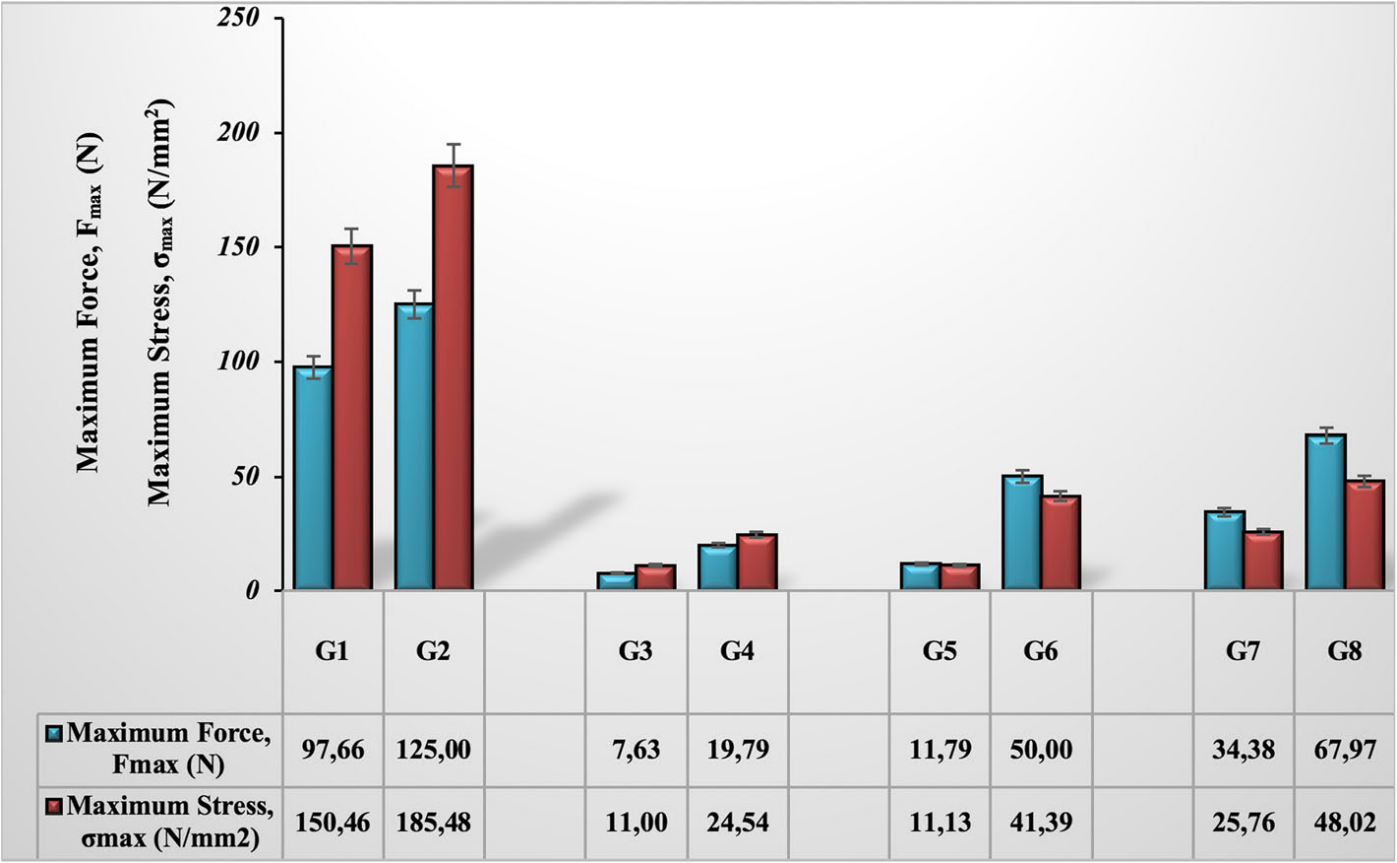
Maximum force and maximum stress values

Figure 8 shows the maximum force and maximum stress values obtained as a result of the 3 PB test as a measure of fracture healing. When the results of the bending tests on no fracture introduced bones (G1 and G2) were evaluated, it was seen that GSPE provided a significant increase in bending strength. On the 10th and 20th days in the groups not given GSPE (G3 and G5), there were no significant union and therefore a significant force (7.63 N, 11.79 N, respectively) and tension relative to force (11 N/mm^2^, 11.13 N/mm^2^, respectively) could not be measured. On day 30 (G7), some improvement was observed and *F*_max_ (34.38 N), and σmax (25.76 N/mm2) values were calculated. On the other hand, it was determined that the bending strength (σmax 24.54 N/mm^2^) obtained on day 10 in the GSPE given group (G4) was almost equal to the bending strength (σmax 25.76 N/mm^2^) obtained on the 30th day of the group which were not given the GSPE (G7). The effect of GSPE was more pronounced with time during recovery. Considering the 30th day that we could say that there was healing, we could see that grape seed extract increased the bending strength of the fractured bone approximately two times (Fig. 8).

**Fig. 9:**
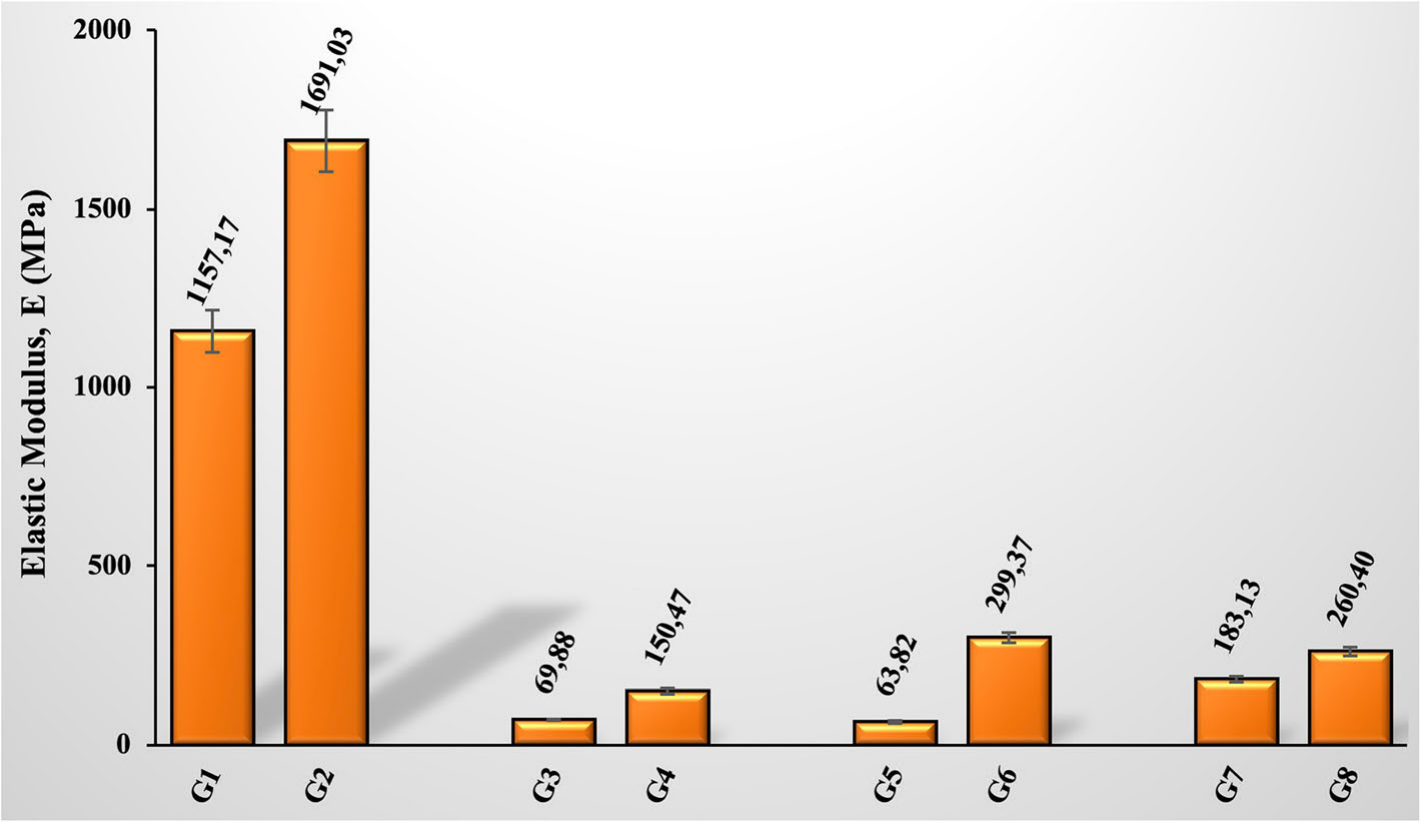
Elastic modulus values

The change in the elastic modulus values of the groups provided similar results to the other changes. On the 10th and 20th days, no significant union was observed in the fracture site in groups not-given GSPE, so we evaluated the recovery period of 30 days. It was calculated that the value of the elastic modulus in the fracture area of grape seed extract given group (G8) increased by about 42% compared to not given group (G7) (Fig. 9). An increase in the elastic modulus value means that the bones supplemented with GSPE can carry higher stresses when exposed to the same deformation amounts in the fracture area.

## Discussion

Fracture healing is a complex but well-regulated process that results in the best possible way of repair of the skeleton and the restoration of its functions [17]. However, there may be some problems in repairing process, and this may adversely affect fracture healing [17, 18]. Non-union of a fracture that is mechanically stable and treated in accordance with fracture fixation methods is most likely related to deteriorated biological processes on the fracture site [17]. One example of these biological deteriorations is an oxidative stress injury [3]. Reperfusion of tissue after transient ischemia caused by damage to the bone after fracture formation may cause free oxygen radicals. Circulating inflammatory cells and osteoclasts play a vital role in the formation of free oxygen radicals [19]. Oxidative damage may occur in the bone when endogenous antioxidant defense systems are insufficient [3]. It is known that antioxidants are useful to eliminate the adverse effects of free oxygen radicals on bone healing [2]. Erdemli et al. [20], in the experimental study which they investigated the changes in liver tissues caused by oxidative damage in rats with fractured mandible, found that using GSPE food supplement reduces oxidative stress in rat liver. Osteonecrosis is one of the bone pathologies in which oxidative damage plays a major role [5]. Song et al. [5] investigated the effects of GSPE on steroid-induced osteonecrosis and found that GSPE reduces oxidative damage and apoptosis, and consequently reduces the incidence of steroid-induced osteonecrosis by 70%. In our study, as a result of histological examinations, we observed that bone healing in rats treated with GSPE was more prominent. One of the reasons for this is that GSPE is a powerful anti-oxidant and by stabilizing free radicals in the environment can induce calcium absorption and fracture healing [21]. Another reason for GSPE, a flavonoid, to stimulate bone healing is possibly due to its phytoestrogenic effect. It is known that calcium intake, together with flavonoids such as ipriflavone, which inhibit bone resorption, has important effects on bone formation [8, 22–24]. Ipriflavone, a derivative of isoflavone, one of the phytoestrogens, has no classical estrogenic effect on the uterus and other female reproductive organs, but it is known to inhibit bone resorption by a mechanism similar to estrogens [25]. Ipriflavone has also been reported to share the release effect of estrogen in calcitonin so that at least part of the antiresorptive effect of ipriflavone can be explained by the release of endogenous calcitonin [26]. Hohman and Connie [11] investigated that the long-term effects of grape-enriched diet on bone in ovariectomized rats, and they concluded that consumption of grape products could improve calcium utilization and suppress bone turnover which may improve bone quality.

The radiological results in our study were consistent with the histological results. There was a significant increase in the Lane-Sandhu [15] radiological scores and fracture callus volume of the GSPE given groups. This callus volume increase may be due to the inhibitory effect of GSPE on osteoclasts as well as its stimulating effect on osteoblasts [4, 27]. Zhu et al. [12] showed that proanthocyanidins inhibited osteoclasts via nuclear factor-kB (NF-kB) and c-Jun N-terminal kinase (JNK) signaling pathways in ovariectomized rats and consequently reduced bone mass loss, in the study which they investigated the direct effects and molecular mechanisms of proanthocyanidins on osteoclastogenesis. In our study, 3 PB tests were performed to evaluate the robustness of the healing bone. The biomechanical strength of the GSPE given groups was significantly higher. These biomechanical results may be explained by which GSPE reduces bone resorption, stimulates new bone formation, and also regulates bone mineralization, which is important for bone strength [10].

This current study has some limitations. First, this was an animal study, the results of which may differ when applied to humans. The second of these limitations is that the effects of GSPE could not be analyzed biochemically in this study. However, the use of GSPE is known to reduce oxidative stress markers in the body [28]. The third limitation is the use of the bending test only for biomechanical testing. Torsion and compression tests would undoubtedly provide valuable information about the biomechanical strength of the healing bone, but according to Oksztulska et al. [29], the most useful method for biomechanical measurements in small bones is the 3 PB test.

## Conclusion

In conclusion, this current study showed that GSPE, a potent antioxidant, has positive effects on bone healing and increases the mechanical strength of the healing bone. Along with this, the biomechanical resistance of the intact bone was significantly increased when supplemented with GSPE. These results indicate that GSPE may be an effective therapeutic agent on bone health and fracture healing.

## Data Availability

The datasets used and/or analyzed during the current study are available from the corresponding author on reasonable request.

## Abbreviations

3 PB: 3-point bending
A: Cross-sectional area
AP: Anteroposterior
DICOM: Digital imaging and communications in medicine
E: Elastic module
GSPE: Grape seed proanthocyanidin extract
I: Moment of inertia
m: Medullary diameter
Mb: Bending moment
ML: Mediolateral
W: Strength module
δ: Deflection
σ_max_: Maximum stress
